# Poor-Law Nursing in England. Poor-Law Nursing To-Day.—II
*The previous article appeared on June 16, p. 211.


**Published:** 1917-06-23

**Authors:** 


### THE MATRONS' AND SISTERS' DEPARTMENT.

### POOR-LAW NURSING IN ENGLAND.

### Poor-Law Nursing To-day.—II.*

The Poor-Law nurse to-day forms a considerable part of the nursing profession. In the separated infirmaries the conditions of training are now very good. The same may be said of the large unseparated infirmaries. In both there is a three years' training, which follows, as far as possible, the lines in large general hospitals.

The class of women applying as probationers is much the same as in other hospitals. Some twenty years ago infirmaries were much understaffed; but now the staffs have been greatly increased, and the hours of work and of off-duty time compare favourably with those of general hospitals. Although the conditions of training differ from those in general hospitals in some respects, the training is as good in its own way, and certainly gives more variety in nursing experience. The medical work is excellent, and includes all varieties of acute cases, as well as phthisis and incurable cases.

The nurse gains experience in the treatment of all forms of children's disease, and also in eye and skin cases. Midwifery training is given, under the best conditions and free of expense, except for the examination fee, in most of the large infirmaries. Another advantage is the nursing of convalescent cases, as patients can remain in infirmaries till recovery is complete, instead of being discharged as soon as the acute stage is over.

The surgical work is much less than in a general hospital, and this is a decided disadvantage. Still, there is generally sufficient to train on, and nurses attend all operations and are carefully taught how to prepare for them. The operating theatres are up to date, and the most modern methods are used. As there is no medical school attached to a Poor-Law infirmary, the nurses have the advantage of doing all the work usually done by students.

All infirmaries have a large number of chronic sick, which is sometimes considered a drawback and is rather monotonous work. But it is experience which proves most valuable to those who take up district or private nursing. The management of helpless patients is often better understood by an infirmary nurse than by those trained in general hospitals.

A Poor-Law probationer has this training in her first year, instead of the amount of cleaning and polishing work that falls to the lot of a first-year probationer in hospital. The theoretical teaching is good. There are regular courses of lectures and class-work from the time the nurse enters her training school. Invalid cookery and massage are also taught now in most large infirmaries. At the end of the three years' course the nurse must pass an examination before receiving her certificate.

This course of training is followed in all large infirmaries, whether separated or unseparated; but the great difference between the two classes is that a separated infirmary is under a medical superintendent, and the unseparated is under the workhouse authority. Though the training may be equally good, and the certificates of equal value, though the number of beds may be larger in unseparated infirmaries and the work better than in some of the separated ones, the loss of position is a very serious matter. The infirmary is under the master, and is a department of the workhouse. It has no separate stores, laundry, or administrative office; in fact, no independent existence.

This unfortunate arrangement was made, in the first instance, to save expense, and this is still the reason given for continuing the system. But separation would only be an initial expense, as infirmaries, once separated, would be more economically managed under a good medical superintendent and infirmary matron who have special knowledge of hospital requirements, and to whom the interests of the infirmary would be the first consideration.

To the master, the workhouse naturally comes first, and he has little or no interest in the infirmary. His experience does not fit him for the position of head of a hospital, for which skilled knowledge is required. The lack of this knowledge leads to bad administration, which is always expensive, and also to difficulties with the medical staff.

When there is no medical superintendent the young resident doctors, though nominally under the master, have practically a free hand and can order anything in the way of diet, treatment, and extras that they like. Naturally, the last thing they think of is expense, and it is difficult to make the nursing staff careful in avoiding waste and extravagance in dressings or other supplies, when the example and influence of the medical men are always on the opposite side. The medical officer of the workhouse, who is non-resident and generally holds other appointments, has no time to attend to these details. He cannot give more than a general supervision to the whole of the workhouse, which contains the imbecile and epileptic wards, as well as the infirmary, and often numbers between two and three thousand beds. Another serious defect is the fact that there is no separate laundry or stores for the infirmary. The lady superintendent is responsible for the ward linen, but she has no control over the laundry, which is entirely managed by the workhouse matron. It is very easy for linen to be lost, stolen, or badly torn; but investigation is extremely difficult, as the laundry superintendent and the ward sisters throw the blame on each other. If the infirmary had a separate laundry the lady superintendent

* The previous article appeared on June 16, p. 211.

would have the supervision of it and could check carelessness on both sides.

The clothing stores are also at the workhouse, and everything required in the wards must be requisitioned for each week. Frequently the articles most wanted are not ready. Either the material has run out or has been used for some other department, and the stock of clothing in the wards cannot be kept up. This would not occur if the lady superintendent could order the materials required and have them made up as they were wanted in the wards. In these and in other ways the separation of the infirmary from the workhouse would lead to economy and efficiency, and, most important of all, the entire staff of the infirmary would be placed under professional control, and be free from the jurisdiction of the workhouse and all that it represents.

